# A feasibility study for a unified, multimodal analysis of online information foraging in health-related topics

**DOI:** 10.12688/openreseurope.16119.1

**Published:** 2023-06-20

**Authors:** Szilvia Zörgő, Gjalt-Jorn Peters, Anna Jeney, David Williamson Shaffer, Andrew R. Ruis, Rik Crutzen

**Affiliations:** 1 Care and Public Health Research Institute, Maastricht University, Maastricht, Limburg, 6200 MD, The Netherlands; 2 Theory, Methodology & Statistics, Faculty of Psychology, Open University of the Netherlands, Heerlen, PO box 2960, 6401 DL, The Netherlands; 3 Baltic-Black Sea Regional Studies Programme, Ivan Franko National University of Lviv, Lviv, Ukraine; 4 Department of Educational Psychology, University of Wisconsin-Madison, Madison, Wisconsin, USA

**Keywords:** methodology, unified methods, data visualization, open source software, multimodal data, digital health literacy

## Abstract

**Background**: Digital health literacy (DHL) is the ability to find, understand, and appraise online health-related information, as well as apply it to health behavior. It has become a core competence for navigating online information and health service environments. DHL involves solving ill-structured problems, where the problem and its solution are not clearcut and may have no single answer, such as in the process of sensemaking. We employ and expand on information foraging theory to address how experts and novices in information retrieval perform a search task. Our overarching aim is to pinpoint best practices and pitfalls in understanding and appraising health-related information online to develop a digital intervention to increase DHL and critical thinking.

**Methods**: In this feasibility study, we recruited a total of twenty participants for our expert and novice subsamples. We collected sociodemographic data with a self-developed survey, video data through an observation protocol of a 10-minute search task, as well as audio-video data via a retrospective think-aloud. The three, multimodal data streams were transcribed and aligned. Codes were developed inductively in several iterations, then applied deductively to the entire dataset. Tabularized, coded and segmented qualitative data were used to create various quantitative models, which demonstrate viability for the qualitative and statistical comparison of our two subsamples.

**Results**: Data were visualized with Epistemic Network Analysis to analyze code co-occurrences in the three aligned data streams, and with Qualitative/Unified Exploration of State Transitions to examine the order in which participants in our two subsamples encountered online content.

**Conclusions**: This paper describes our methods and planned analyses elaborated with mock figures. Quantifying qualitative data, aligning data streams, and representing all information in a tabularized dataset allows us to group data according to various participant attributes and employ data visualization techniques to pinpoint patterns therein.

## Introduction

### Digital health literacy

Since the creation of Web 2.0, the internet is characterized by profuse user-generated content and participatory culture. This emerged in tandem with novel avenues for information production and fundamentally changed the information landscape in general, as well as in specialized domains, such as health and health care. Currently, more than half of EU citizens aged 16–74 report regularly seeking health-related information online ^1^, despite the fact that the internet is an information ecosystem riddled with contradictions and inaccuracies ^2^. Digital health literacy (DHL), the ability to find, understand, and evaluate health information in digital environments and apply it to health behavior ^3, 4^, has become a core competence for navigating online information and health service environments. DHL plays a crucial role in individual decision-making, which, as the COVID-19 pandemic has exemplified with respect to vaccination and other preventive measures, also greatly influences public health and collective well-being ^5^.

DHL is associated to both health and many of its determinants ^6^. Higher levels of DHL has been found to correlate with better health outcomes and desirable health behaviors, such as prevention and management of chronic disease ^4^. DHL is also associated with the sustainability of the health care system through better health outcomes ^7^, with the potential to reduce disparities affecting disadvantaged and underserved groups ^8^ by promoting health equity with tailored, patient-facing digital tools. Vulnerability to low DHL is most common among populations with higher age, lower socio-economic position, underserved neighborhoods, and degraded physical environments ^2^. Understanding what determines DHL is essential to design interventions to improve DHL, disseminate public health information, and for policy development on the future of digital health platforms and services ^5^.

Like DHL, the broader constructs of Health literacy is a formative construct that itself consists of four constructs: ability to 1) access or obtain, 2) understand, 3) appraise, and 4) apply information relevant to health, which manifest in three domains: health care, disease prevention, and health promotion ^2^. These constructs are also generally employed in instruments to measure DHL. Past studies on DHL have primarily utilized self-reported questionnaires, which may rely on the respondent’s own assessment of their health literacy as applied to digital contexts. Questions such as “ *When you searched the internet for information about the coronavirus or related topics, how easy or difficult was it to…*?” may aid obtaining estimates of an individual’s confidence in their own skills, but as studies in other areas have shown, self-assessment requires considerable metacognitive skills, often failing to provide valid measurements of the target construct ^9^. Observations of users utilizing their DHL in an online environment may complement self-assessment in a profound way, enabling a deeper understanding of how individuals engage with and interpret health-related information in digital environments.

The observation of user behavior regarding health-related information online has chiefly been limited to well-structured problem-solving ^10^: tasks that require the application of a finite number of concepts and principles and consist of a well-defined initial state and goal state ^11^. For example, single-site usability studies restrict user navigation to a specific website and often require finding one particular piece of information (i.e., there is one correct solution to the problem). Results from such studies convey information on a participant’s ability to retrieve specific information and the usability of a particular website, but do not lend much insight into organic search behavior or how users solve problems with multiple or unclear solutions, that is, ill-structured problems. Ill-structured problems generally do not have one correct answer, require the integration of several content domains, contain problem elements that are unknown, and possess multiple solution paths and sets of criteria for evaluating solutions ^11^. In practice, engaging with health-related information involves solving both well-structured and ill-structured problems; our study aims to shed more light on the less-studied processes of ill-structured problem-solving connected to the DHL dimensions of *understanding* and *appraising information* relevant to health.

### Theoretical framework

Our theoretical framework is provided by information foraging theory, which describes how people search for information (online) and how they navigate among sources of information ^12^. Information foraging theory stipulates that people “forage” for information as animals forage for food. An individual’s information environment consists of a combination of 1) stimuli (verbal, visual, etc.) that are perceptually accessible (e.g., text in a book, a video on a smartphone, a diagram on a website, the speech of a person they are listening to) and 2) opportunities for interaction that would make presently unperceivable information perceivable (by e.g., scrolling down on a webpage, clicking on a button or text link on a website; taking a book off of a shelf; approaching a person with a question). In addition, information environments are “patchy” because information is clustered in certain areas, for example, on bookshelves, in libraries, on websites, and in databases ^12, 13^. As information foraging theory does not specify a clear definition of a patch, for the purposes of our study, we define patches as physical or virtual environments where produced and/or curated information can be stored and potentially accessed by users. This definition allows for patches to be operationalized as, for example, entire libraries or single books, as well as databases or individual webpages, depending on research objectives.

Individuals generally engage with their information environment to achieve an information goal, which we define as a presently active goal a person has to obtain declarative knowledge about the world (e.g., how many inhabitants the village of Etenaken has; whether face masks protect against COVID-19 infection). We use the term “active” to distinguish and explicitly exclude latent information goals ^1^, where an active goal requires that a person has to have decided to invest resources in achieving the goal, a latent information goal refers to curiosity or intent in the absence of goal-oriented behavior (e.g., a person may in principle want to know more about a topic but never invest in obtaining relevant knowledge).

Information foraging theory stipulates that a user’s navigation in and among information patches depends on “information scent”; a high information scent leads to foraging behavior that aids the user in achieving an active information goal ( 12 p72). We define information scent as a person’s representation of the extent to which investing in a given patch of their information environment will aid progression toward a given information goal. This definition places emphasis on both the user and stimuli in the information environment. Previous descriptions of information scent, such as that of Pirolli *et al*., focus more on the stimuli: users engage with environmental stimuli (e.g., textual links) that are assumed to have “the maximum expected utility” ( 12 pg 76) in achieving an information goal, but this understanding of information scent does not easily accommodate varying user representations of utility or meaning. This also implicitly suggests that patches and particular stimuli in patches *have* information scent ^2^, and therefore, information scent can be accredited to the stimuli alone: e.g., certain textual links “have high information scent” compared to others ^3^ ^14^. In our definition, such information scent would always be a function of both stimuli and stimulus attributes as well as factors influencing a person’s processing of those stimuli in light of their active information goal(s). Although the authors also describe information scent as contingent on the user’s ability to detect and engage with stimuli ( 12 pg 68), the component in their model that symbolizes user representations is uniform across individuals and time (see below).

In work thus far, chiefly by Pirolli *et al.* ^14, 15^, information scent has been computed with “semantic similarity” (proximity of words within documents in a corpus) with Natural Language Processing techniques, such as Latent Semantic Analysis and Latent Dirichlet Allocation. A chosen corpus and the derived word proximity scores form what is referred to as a “scent database”, which, in turn, serves as the basis of computing information scent. Typically, researchers utilize a large textual database (e.g., a corpus containing thousands of news articles) that is not tailored to the population under study. Proximity scores are considered uniform across users who share an information goal (scores are derived from associations between words in the task description and textual environmental stimuli, specifically, links and hyperlinks) and across an entire task (score computation does not change over time).

Given our definition of information scent, operationalizing information scent using proximity scores in this way is not possible, since these cannot incorporate user characteristics. In our definition, patches in themselves cannot have information scent: instead, they have attributes that contribute to information scent, but with varying degrees depending on users’ extant internal representations (among other things). Similarly, information scent is not an attribute of an individual: it can vary in the same person over time and context, depending on which information goals are active at any given time, the patches they are exposed to, and the knowledge they have accumulated over a task. One implication of this definition is that different people will have different information scent values for the same patch. Another implication is that as a person progresses towards their information goal, the information scent of a given patch can increase or decrease depending on how acquired knowledge influences their processing of their information environment. In addition to modifying the definition of information scent, we propose the following modifications to information foraging theory:

*Code co-occurrences instead of semantic similarity*
  We employ code co-occurrences instead of semantic similarity to compute information scent. Codes are labels attached to data fragments that denote the observation that that fragment expresses a construct of interest. Owing to their conceptual nature, we assume that codes are more adequate at capturing meaning than individual words alone, and therefore that they may be employed to create more accurate models of the data. It is the co-occurrence of these codes within designated segments of data that serves as a basis for computing information scent. This requires codes to be applied deductively to ensure equivalent application across the dataset.
*Sampling from the target population*
  We sample data from the target population, as opposed to using data from newspaper articles or similar sources.
*Differences between individuals*
  Information scent in previous studies has been considered uniform across a sample ^14^, but we attempt to develop a process through which it can be tailored to an individual or subsample to reflect various representations. Because co-occurrences can vary according to mental models, we hereby take into account that various populations and individuals may have different association strengths.
*Differences over time*
  As currently operationalized, information scent does not evolve over time (e.g., during the completion of a task). Yet, as a user forages for and encounters information, their representations may also change, which should be reflected in information scent values as well. To accommodate this assumption, we propose an evolving model of information scent, from a baseline state through different states at various points in the task to an end state.

Theoretical definitions and modifications can be summarized in the following auxiliary methodological, ontological, and epistemological assumptions. First, contextualized interpretation is necessary for effective hermeneutics. Second, words as the smallest unit of analysis do not present sufficient context because most relevant constructs cannot be identified with single words. We assert that word co-occurrences are insufficient for identifying and describing meaning within qualitative data, but that meaning can be captured in codes. We assume codes can be systematically applied to qualitative data, and that association strengths can be computed from code co-occurrences. We posit that association strengths computed from code co-occurrences are a viable alternative to semantic similarity in calculating information scent, and that corpora collected from specific populations can serve as the basis of a scent database. For more on the theoretical framework and problematization, see: Problematization available at: https://osf.io/5kw7n).

### Objectives and research questions

This study is conducted under the aegis of a Marie Skłodowska-Curie post-doctoral fellowship called “Smart Online Searching to Improve Patient Safety” (SOS-TIPS), which aims to explore how people navigate health-related information online. The overarching aim of SOS-TIPS is to develop an intervention to increase information and digital health literacy as well as scaffold critical thinking. The present text explicates the design of a feasibility study that will explore the how novices and experts in information retrieval and assessment appraise and understand health-related information online. We chose to compare experts and novices in order to better understand best practices and pitfalls in solving ill-structured problems regarding health-related topics, and more specifically, to see how assessing the relevance and trustworthiness of (a source of) information differs between experts and novices, as well as to compare their abilities to understand encountered information that contributes to their opinions on a given topic. This feasibility study will be followed by a larger, multi-site study. Our research questions are as follows:

RQ1: How do novices and experts describe the online content they interact with during an information seeking task?
RQ2: How do participants’ perceptions of their understanding of the researched topic evolve over the duration of the search?
RQ3: What sources of information are explored during the task?
RQ4: What patch features are utilized to assess the trustworthiness of (sources of) information?
RQ5: How does information scent differ between novices and experts?

## Methods

Ethics approval was gained from the University of Wisconsin-Madison Institutional Review Board. The study (2022-0588) was determined to meet the criteria for exempt human subjects research as defined under 45 CFR 46:(3)(i)(B). Written informed consent to participate in the study and make their anonymized data public was obtained from participants in a pre-interview survey. In accordance with open science principles, our entire project is publicly accessible in our Open Science Framework repository, available at https://osf.io/ynt7a.

### Sampling considerations

**Figure 1.**
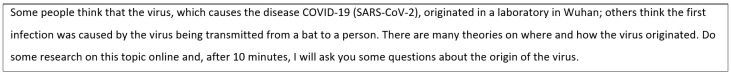
Description for online search task.

We included two populations of internet users in our study. The first population consists of experts in information retrieval and assessment: individuals who have educational or work experience as librarians, journalists, or related professionals. The second population consists of lay people who are not experts in information per this definition. We sampled from these two populations, respectively called: “Expert subsample” and “Novice subsample”. Our general sampling strategy was purposive, and we aimed for homogeneity within and across subsamples with respect to the following criteria:

Location (United States, Wisconsin) – As we investigated online searching behavior, and geographical location is a marked influencing factor in the content of and access to information ^16^, we wanted to keep location constant (within the state).
Age (range: 18 – 39 years) – As age is a known variable affecting computer skills and information literacy, we set an age range (designated as “younger” in a three-tiered classification) that offers some homogeneity in this factor ^17^.
Language skills (no Chinese language skills) – As completing the information goal within our study (see Figure 1 for task description) may be greatly influenced by prior knowledge of local events in China since 2019, which we assumed is correlated with a working knowledge of the Chinese language, any speakers of Chinese were excluded from the study
Ethnicity (non-Chinese) – We also assumed that being of Chinese descent signifies the same biasing advantage to performing the designated task within the study as does speaking the language. Thus, we excluded people of (self-reported) Chinese descent as well. Descent was operationalized as “born in China” and/or “child of Chinese parents” (either or both parents born in China).

Thus, our inclusion criteria were: resident of the state of Wisconsin (USA), aged 18–39 years, speaker of English; our exclusion criteria were: not a resident of Wisconsin, outside of age range, speaker of Chinese (Mandarin), of Chinese descent.

We included ten participants in both subsamples (N=20 in total), which provided us with enough participants for various possible post-data collection groupings, not only the specified subsamples. This sample size was adequate for the study because 1) for a feasibility study, a proof of concept in terms of our framework and tool, a relatively small number of participants suffices, and 2) we utilized manual coding for all data types (to map processes and discover challenges involved) and had to plan realistically in terms of what we are capable of curating and coding within our timeframe. In a subsequent project, we will conduct a larger-scale study with more participants and be able to automate several processes (e.g., aspects of data collection, segmentation, coding).

### Data collection

*Recruitment*

The expert subsample was recruited via email from the University of Wisconsin-Madison Information School, as well as Departments: Library and information studies and Journalism. The novice subsample was recruited via a university listserv. Recruitment information and screening procedures are available in our repository. Participants were compensated for their time with a $30 Amazon gift card.

*Data collection instruments*

We used observational and think-aloud protocols to collect data on information foraging within the framework of an interview. Our observational protocol contained the specifics of observing online foraging in a 10-minute search task with the information goal of learning about various COVID-19 origin theories; the task description is displayed in Figure 1.

**Figure 2.**
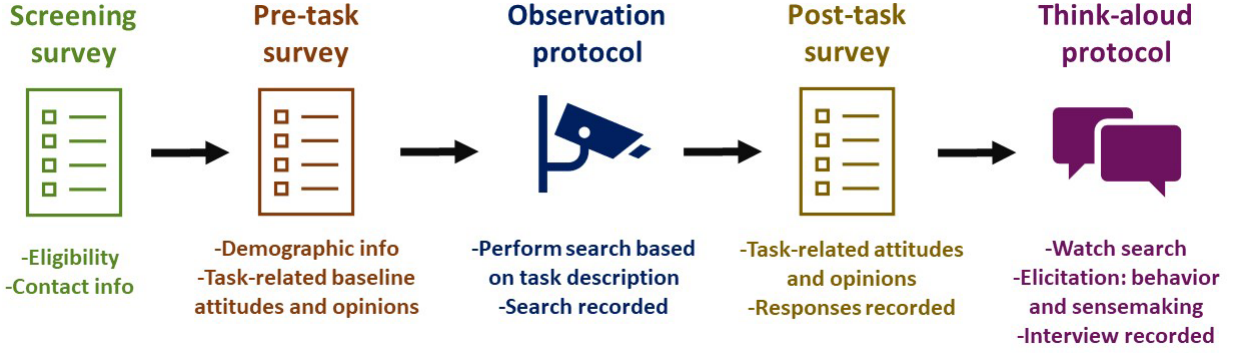
Process of data collection.

The think-aloud protocol standardized how self-reflection on behavior was elicited, namely, questions to pose when the participant enters a patch, leaves a patch, or engages with content within the patch. Questions probed general impressions and indicators of trustworthiness for all visited websites, as well as why participants engaged and disengaged with them. Both protocols were conducted online via a video conferencing platform. Additionally, we employed three surveys: a screening survey, a pre-task survey, and a post-task survey. The screening survey contained items used to evaluate participant inclusion. The pre-task survey was administered asynchronously within 48 hours of the scheduled interview appointment to collect basic sociodemographic data. The post-task survey was conducted synchronously to record the response to the information goal via verbal elicitation immediately following the search activity. All data collection instruments are available in our repository. Figure 2 displays the process of screening and data collection.

The observation phase of data collection yielded a 10-minute video (no audio) during the online search task. The think-aloud yielded audio-video data of varying durations (mean: 65 mins; range: 35–111 mins). Open and closed-ended questions in the two surveys yielded both numerical and textual data.

### Data curation and coding

*General goals*

Our data curation aim was to represent data from our four data sources (two surveys, transcribed video, transcribed audio) in a single, tabular dataset. This process served to merge data sources and, congruently, data from our two modalities (video and text), resulting in a dataset that contains both segmented qualitative data and the codes applied to each segment. We refer to the latter as the quantified qualitative data, and we use this to more easily identify patterns within the entire sample and subsamples, as large amounts of qualitative data may often inhibit seeing general tendencies and drawing conclusions for samples or subsamples. With that final dataset, we can create models of our data to answer research questions and display complex results in an effective and visually intuitive manner. We assert that such quantitative models shed light on prevalent patterns in rich data, and in turn, a qualitative understanding of the data advances model development and interpretation. The qualitative and quantified aspects of the data can mutually inform each other and facilitate the generation of warranted claims.

*Employed standard*

We are using the Reproducible Open Coding Kit (ROCK), a standard which enables transparent qualitative research and is primarily implemented in the R package rock. The ROCK helps researchers organize data in plain text files (i.e., sources), specify characteristics of data providers, data provision, or data (i.e., attributes), code and segment narratives, as well as perform various analyses. In accordance with the ROCK standard, the R package allows making these steps explicit and transparent, thus improving transparency, inclusivity, and accessibility of research while minimizing research waste. Furthermore, the ROCK facilitates sharing coded qualitative data, enabling other researchers to reproduce the coding process, compare results, and collaborate by sharing or expanding the coding system. To read more about the standard and its application, see: 18.

*Operationalizing information patches and information scent*

To investigate online organic search activity, we have operationalized patches as any type of file that a browser can render when a user visits a particular URL. This included search engines, stand-alone pdfs, and pictures viewed on a separate page. Patches are the objects to which both behavior (human-computer interaction [HCI]; what participants did on a website) and cognition (narratives; what participants said regarding a website) were aligned, and thus form the basis for data stream alignment as well as higher-level segmentation (see: below).

Earlier, we defined information scent as a person’s representation of the extent to which investing in a given patch of their information environment will aid progression toward a given information goal. In our study, since the goal was to research COVID-19 origin theories and formulate an opinion, this utility was operationalized based on the health literacy constructs *ability to understand* and *ability to appraise* health-related information. Accordingly, information scent in this study concerns the participant’s assessment of a patch regarding its *relevance to the topic*, its *trustworthiness*, and its *ability to contribute to understanding* the topic. High information scent hence is the ability to pinpoint indicators of the relevance and trustworthiness of a patch, and be able to gather information from a given patch. These three components of information scent are defined below:

*Relevance* is defined as the participants’ estimation of a patch’s usefulness in addressing the topic at hand and in achieving the information goal.
*Trustworthiness* is defined as the participants’ estimation of the veracity of patch content and/or the credibility of content creator(s) via pinpointing physical features of patches.
*Understanding* is defined as the participants’ ability to explicitly reflect on the substantive aspects of patch content, regardless of whether they agreed with the content or whether it formally contributed to their opinion.

*Survey data*

Survey results were downloaded from the platform (Qualtrics) in a .csv file and reformatted in YAML, a human and machine-readable markup language that can be processed with rock. Our final attribute list contained values for all survey variables for each of our participants. The coding for survey responses is available in our repository. ^4^ Table 1 contains the attributes recorded for each data provider (i.e., case).

**Table 1.** Case attributes.

A ttribute	D escription / S urvey Q uestion	E xamples	A ttributes in YAML
Case identifier (caseID)	Unique label containing the ordinal numbering of participants	case_001, case_002	--- ROCK_attributes: - caseID: "case_001" groupID: "E" sex: "male" gender: "woman" ethnicity: "no_hisp" race: "white" country: "USA" education: "SC" pol_aff: "NA" - caseID: "case_002" groupID: "E" sex: "male" gender: "man" ethnicity: "no_hisp" race: "black" country: "NGA" education: "BA<" pol_aff: "other" ---
Group identifier (groupID)	Self-identified, non-unique label assigned to participants upon completion of screening survey	Expert, novice	
Sex (sex)	What sex were you assigned at birth, on your original birth certificate?	Male, female, intersex	
Gender (gender)	How do you currently describe your gender?	Man, woman, transgender	
Ethnicity (ethnicity)	What is your ethnicity?	Not Hispanic or Latino, Hispanic or Latino, other	
Race (race)	What is your race?	American Indian or Alaska Native, Black or African American, White, Asian	
Nationality (country)	In what country were you born?	ISO 3166-1 alpha-3 country codes, e.g., USA	
Level of education (education)	What is the highest level of education you completed?	High school or equivalent, Bachelor’s degree or higher	
Political affiliation (pol_aff)	What do you consider your political affiliation?	Democrat, Republican, Independent, Liberal, Conservative, Other	

*Think-aloud data*

Audio data from the think-alouds were transcribed in an automated process with Otter.ai, manually corrected for accuracy, and placed into separate plain text files for each data provider. ^5^ Transcripts were anonymized for personal and/or identifying information. Our analyses (see: below) require us to segment our data, that is, divide them into meaningful parts ^19^. In the think-alouds, the smallest meaningful pieces of our data (i.e., utterances) were defined as sentences; these are delimited by newline characters (thus sentences fall on separate lines) and are given unique utterance identifiers (UIDs). Coding is performed on this level of segmentation.

We are currently developing codes for the think-alouds, which address patch relevance, trustworthiness, and understanding of content. This will likely yield three code clusters: 1) *Relevance* (factors that contribute to participants’ assessment of patch usefulness in light of achieving the information goal), 2) *Trustworthiness* (factors that contribute to participants’ assessment of patch content veracity and credibility of content creator(s)), and 3) *Understanding* (formulations of substantive aspects of patch content). Our coding process follows the stages below:

1) Free inductive coding performed autonomously by three raters.
2) Triangulation and creation of a tentative codebook.
3) Test coding performed autonomously by three raters on the same subset of data.
4) Triangulation and repetition of steps 2 and 3 until a final codebook is developed.
5) Inter-rater reliability testing (using Cohen’s kappa as indicator) to confirm shared understanding and pinpoint potential discrepancies.
6) Triangulation and repetition of step 5 until Cohen’s kappa ≥0.90 is reached.
7) Application of final code structure to full dataset deductively (coders will “specialize” in certain codes).

All coders will be using a code cluster each ( *Relevance*, *Trustworthiness*, *Understanding*) to code the corpus manually with the Interface for the Reproducible Open Coding Kit (iROCK) ^6^. Code identifiers are specified according to the ROCK standard: the code label is placed in between two pairs of square brackets, e.g.: [[Code_A]], [[Code_B]]. Coders will “specialize” in a cluster to maximize reliability (consistency) in coding. We will measure inter-rater reliability with Cohen’s Kappa ^20^ as we develop our final coding structure because even though coders will be working with different codes, we believe that reaching agreement on what various codes mean and how to apply them is crucial regardless of whether the raters then use the same codes and split the data among themselves or use different code clusters to code the entire dataset. We will also be computing intra-rater agreement with Cohen’s Kappa for all raters after reaching 50% of the dataset (i.e., after interview 10) and after reaching 100% of the dataset (i.e., after interview 20) to test how consistently a single coder applies their code cluster throughout the coding process. All coders will have a coded version of every think-aloud, thus producing 60 coded sources in total, which will be merged by participant. This will result in a merged version of every think-aloud (N=20) containing codes from all three code clusters. Merging is performed with the rock package and is based on the utterance identifiers.

*Video data: Human-computer interactions*

Relevant aspects of video data to be transcribed were: observed HCI and screen content; these were transcribed separately. HCI observations were considered a stream of data and transcribed in a specific template as follows:

 

“Action | Content | Location”

In this template, *Action* refers to observed actions performed in physical engagement with the computer: type, click, scroll, hover, and highlight. *Content* indicates what the action referred to, what it was performed on, or a specification of the action: e.g., the object selected, the characters typed in, or the direction of a scroll. *Location* signifies the place of action within the window, e.g., search bar, page, hyperlink.

Action codes were developed inductively by two coders independently reviewing the entire dataset and triangulating their tentative codes in one round then finalizing the five codes. Content codes were non-categorical and fully inductive as the screen content itself determined this element of our template. Location codes were developed separately for Search Engine Results Pages (SERPs) and websites. SERP codes were developed from the collection of SERP elements as described by Oliveira ^21^; location codes for websites were developed inductively in the same manner as action codes. Each HCI observation was placed on a new line in the file and given an identifier with ordinal numbering (e.g., HCI1, HCI2).

To specify which data is associated with which website, we employ “class instance identifiers” within the ROCK standard; once designated within a source, this type of identifier is automatically applied to each succeeding utterance until a new class instance identifier of the same class (in this case, another patch identifier) is designated. Patch identifiers (cf. Table 2) are added to the data manually, and their format is similar to codes in that they fall within two pairs of square brackets; for example: [[patch_001_1]] designates the first patch in the first interview, [[patch_001_2]] designates the second patch in the same interview. Table 2 contains patch attributes, their description, and their representation in YAML.

**Table 2.** Patch attributes.

A ttribute	D escription	E xamples	A ttributes in YAML
Patch tag	Non-unique label assigned to describe main website content and generator of content	PubMed, CNBC news	--- ROCK_attributes: - patchTag: "UW-Madison Libraries" domain: "library.wisc.edu" pid: "patch_006_1" patchType: "engine" start: "00:00" end: "00:03" - patchTag: "Google All" domain: "google.com" pid: "patch_006_2" patchType: "engine" start: "00:04" end: "00:08" ---
Domain	Second and top-level domain (and subdomain, where applicable)	google.com, ncbi.nlm. nih.gov	
Patch identifier	Unique label containing the case identifier and the ordinal numbering of patches within a case's search	patch_001_1, patch_ 002_15	
Patch type	Categorical value indicating general type/ function of website	Engine, SERP, page, file	
Start time	Start of activity on patch (marked by change in URL)	"00:02"	
End time	End of activity on patch (marked by leaving patch, i.e., closing the tab/window or moving to another one or change in URL)	"00:15"	

Patch tags were fully inductive, based on the URL and/or page title (a complete list is available in our repository). Patch types were developed inductively by two coders independently reviewing the entire dataset, triangulating their tentative categories in one round and finalizing the four categories contained in Table 2. These categories were then applied to designate the attribute value for each patch. For a full description of the attributes, see our Comprehensive Codebook (available at: https://osf.io/hu28c).

*Video data: Screen content*

Some aspects of screen content are not recorded as attributes but are coded directly from the raw video data. The content of patches is not scraped for the feasibility study but will be for future studies. Codes will be adopted from the participant think-alouds, i.e., codes concerning *Relevance*, *Trustworthiness*, and *Understanding*. Screen content for each search task is placed in a separate plain text file, and patch identifiers are applied to distinguish data on each patch.

*Aligning think-aloud and video data*

**Figure 3.**
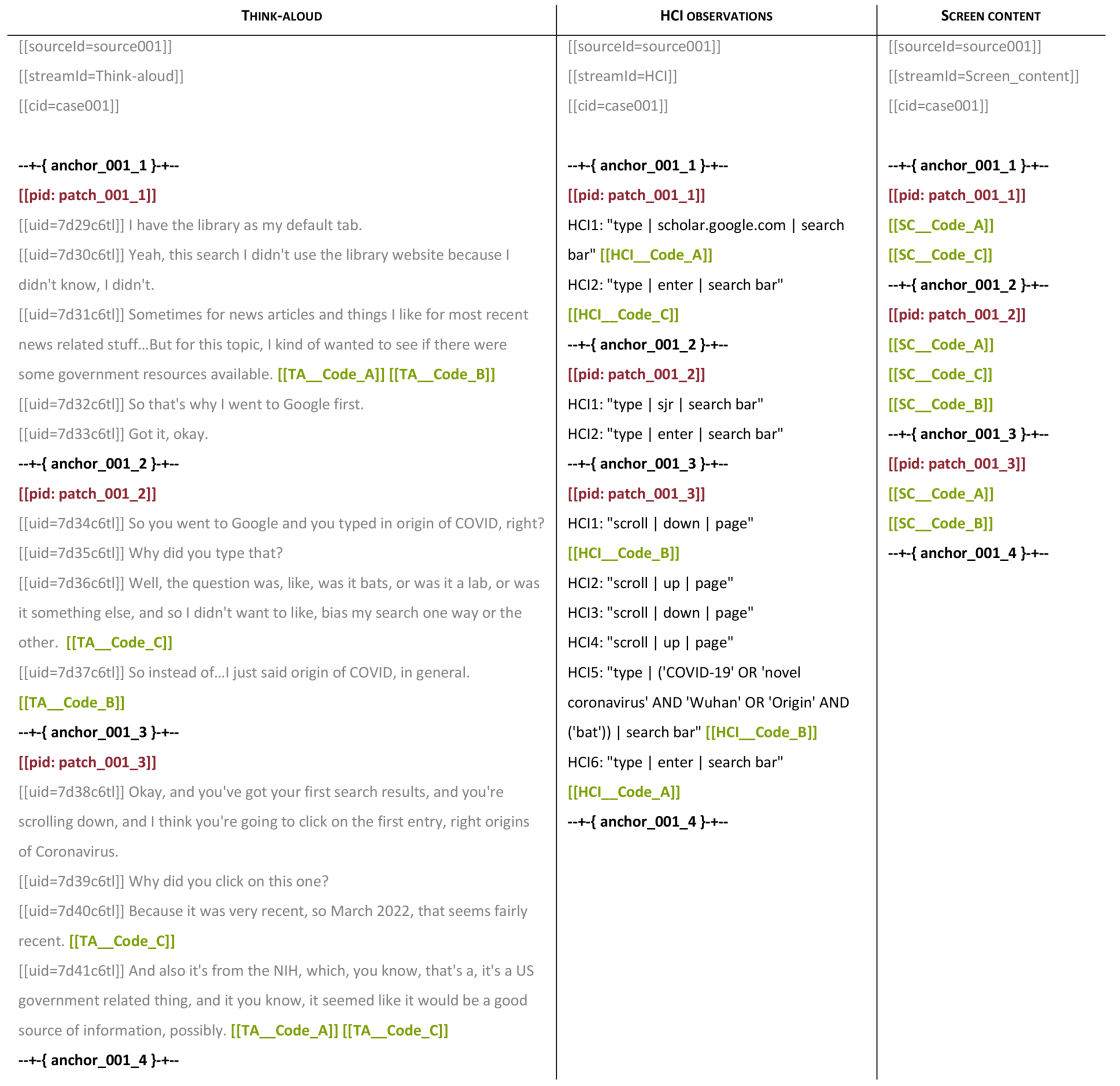
Transcribed data streams with codes, patch identifiers, and anchors.

Information patches function as a way to synchronize our data streams: narratives from the think-alouds, HCI observations, and screen content. To align these streams of data, we embedded anchors in each source. Anchors are text strings that occur in all streams and allow software (in our case, the rock R package) to map codes from all streams onto a primary stream. In each stream of data, we specify unique anchors that indicate in all streams where there is a transition to a new patch and thus where data streams should be aligned. Figure 3 illustrates three sources containing different streams of data for three patches, with codes from a shared coding scheme (green), patch identifiers (red), and anchors (black).

*Creating the unified dataset*

To create a dataset with our qualitative data, its quantified form, and survey data, the coded sources will be parsed with the rock package creating an R object that contains all data from all participants in tabular form. The data from all streams will then be mapped onto one primary stream using the anchors. The resulting data table will be exported as a .csv file. Our unified dataset will contain rows that are determined by our primary data stream: utterances (sentences) in think-aloud data. The other two data streams (HCI and screen content) are aligned based on anchors specifying data corresponding to successive information patches within a source. Columns in this dataset contain original data from the three streams, as well as values for all variables in our study. These variables, if recorded as attributes (for cases and patches) are represented in categorical form. Other variables are our coding, which are represented in a column each in binary form.

### Analyses and modelling

Analyses are organized in correspondence with our research questions (RQ1-5) under the overarching aim of exploring differences between experts and novices in information retrieval and appraisal during an ill-structured task regarding health-related information. Below are our analyses, mock visualizations and their descriptions intended to convey how we envision the final models to look, as well as an explanation of how we intend to use the tools to construct these models.

***RQ1: How do novices and experts describe the online content they interact with during an information seeking task?***

The analysis conducted to answer this RQ is fully based on the coded think-aloud data and will show how encountered online content was interpreted in our subsamples. Our objective is to describe the cognitive connections individuals and groups of data providers make in their narratives. As we are interested in connections, we chose a network paradigm operationalized with Epistemic Network Analysis (ENA) ^22^ to model constructs of interest in the *Understanding* code cluster and the relationship between them.

**Figure 4.**
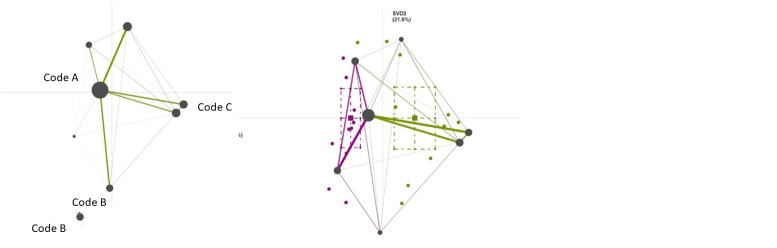
Mock mean epistemic networks for the novice group (purple, left) and the expert group (green, right) and a comparison plot (center) showing the differences between mean networks.

Figure 4 displays mock mean epistemic networks for the novice group (left) and the expert group (right) showing the co-occurrences among qualitative codes in given segments of data. The thickness and saturation of the edges (lines) indicate the relative frequency of co-occurrence between each pair of codes; the size of the nodes (black circles) indicates the relative frequency of each code within that group. In the center is a mock difference graph (comparison plot) showing the subtracted mean networks of the novice group and the expert group. These graphs are calculated by subtracting the weight of each connection in one network from the corresponding connections in another. The thickness and saturation of each line indicates the relative difference between the two groups: purple lines indicate connections with higher relative frequencies of co-occurrence among novice users, and green lines indicate connections with higher relative frequencies among experts.

Describing in detail how networks are computed and generated does not fall within the scope of this paper; for more detailed information, see: 19, 23. Succinctly ^7^, ENA produces a matrix with code co-occurrences and calculates the co-occurrence frequency of each unique pair of codes within a given segment of data. Segments are designated by defining utterances (smallest codable pieces of data) and creating groupings of those utterances to provide meaningful context, that is, “relational context” ^24^. ENA uses two parameters to operationalize relational context: conversation and stanza window. Conversations are groupings of utterances to be connected in a model; for this analysis, we chose information patches as our conversation, as we only wanted to allow code co-occurrences to take place within narratives regarding a given patch and not across various patches. Relational context is also critically determined by stanza window, the chosen mode of co-occurrence accumulation. Stanza window types, sizes, and their effects on network models are described elsewhere in greater detail ^25– 27^.

After ENA computes the cumulative frequencies for each think-aloud segmented according to patches, the cumulative co-occurrence matrix is represented as a vector, and the vectors for all think-alouds together can be considered to form an n-dimensional space. Due to the fact that these vectors may vary in length (because they contain co-occurrences from different amounts of qualitative data), each vector is divided by its length. This normalization of vector lengths captures the relative frequencies of code co-occurrences independent of narrative length and also converts edge weights to fall between zero and one. Subsequently, ENA applies a dimensional reduction procedure (singular value decomposition or means rotation) similar to principal components analysis, to reduce the n dimensions to two dimensions. These two dimensions form the axes along which the vectors (containing the co-occurrences aggregated for each think-aloud) are then projected as points into a two-dimensional space (ENA projection space). These are referred to as ENA scores.

An ENA projection space can be generated for individual data providers and/or groups of participants and offers two coordinated representations of the data: 1) ENA scores, or the position of each network in the two-dimensional space, and 2) Network graphs per participant (or group of participants) in which the nodes represent the codes and the edges depict how the relative frequency with which each pair of codes co-occurred within the specified segments of data. The colored circles in Figure 4 show the ENA scores (network locations) of each novice’s (purple points) and expert’s think-aloud (green points). The colored squares are the mean network locations (mean ENA scores) of each group, and the dashed lines around the means represent the 95% confidence intervals on each dimension.

Because all graphs within an ENA model share a projection space and are thus coordinated representations of the data, the location of nodes in all networks is identical and the location of ENA scores is meaningful: ENA scores in the vicinity of certain nodes represent narratives that are more saturated with co-occurrences between those codes, and similarly, the location of nodes in relation to each other (how the nodes of a network appear in the projection space) carries information about their roles in the corpus. Also, node positions can be used to interpret the dimensions and the locations of the networks on those dimensions.

***RQ2: How do participants’ perceptions of their understanding of the researched topic evolve over the duration of the search?***

In our second analysis, we examine how participants and subsamples described content at each website and model the evolution of their understanding over time. Our objective is to create a patch-by-patch model of the path taken through the search task, in essence, break down the mean networks generated in our first analysis to show individual networks at each patch over time. To create these models, we will be using Trajectory Epistemic Network Analysis (tENA), which enables us to juxtapose the dimensions developed in analysis 1 with patch order.

**Figure 5.**
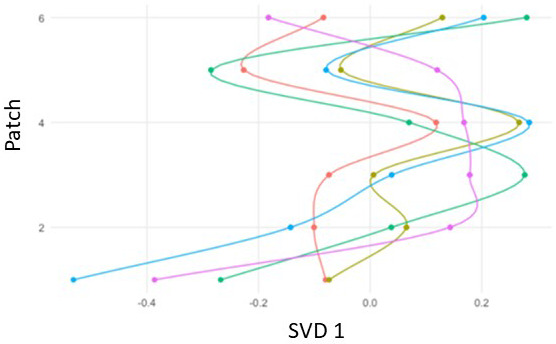
Mock trajectory model in six patches for five cases on the first ENA dimension (SVD1).

Figure 5 shows a trajectory model for five cases over six patches. The ENA space was created in the same manner as in Analysis 1. Both dimensions in the projection space can be used to interpret the differences between networks, and the position of any point is therefore interpretable based on its x and y coordinates. Figure 5 displays differences among cases on the first dimension created with singular value decomposition (SVD).

The tENA projection space is created in the same process as with ENA (see Analysis 1 for description). Both dimensions and the x and y coordinates of any point can be used to interpret the differences between networks. The two interpretable axes can be separated and ordered by patch (or e.g., time spent on patch), making possible the representation of trajectories across both ENA dimensions. In a trajectory model, every ENA score is interpreted by three variables: its x-value, its y-value, and whatever variable is defining the trajectories (e.g., patch, time, duration) ^28^. Visualizations are co-registered, that is, the mathematical properties of a model are aligned with information in associated visualizations. The three variables require three coordinated models: two re-ordered plots and the main ENA space. To accomplish this, two re-ordered plots are generated alongside the original ENA space. Complex temporal information is decomposed in these visualizations to display simultaneous change in pairs of variables, making changes over time easier to interpret.

***RQ3: What sources of information are explored during the task?***

Our third analysis investigates the likelihood of group foraging tendencies by examining the transition probabilities between types of content in a subsample. Our objective is to compute and visualize the likelihood of transition from one content type to another and how much time was allocated to each patch content type within a subsample. To create this model, we are developing a novel tool called Qualitative/Unified Exploration of State Transitions (QUEST).

**Figure 6.**
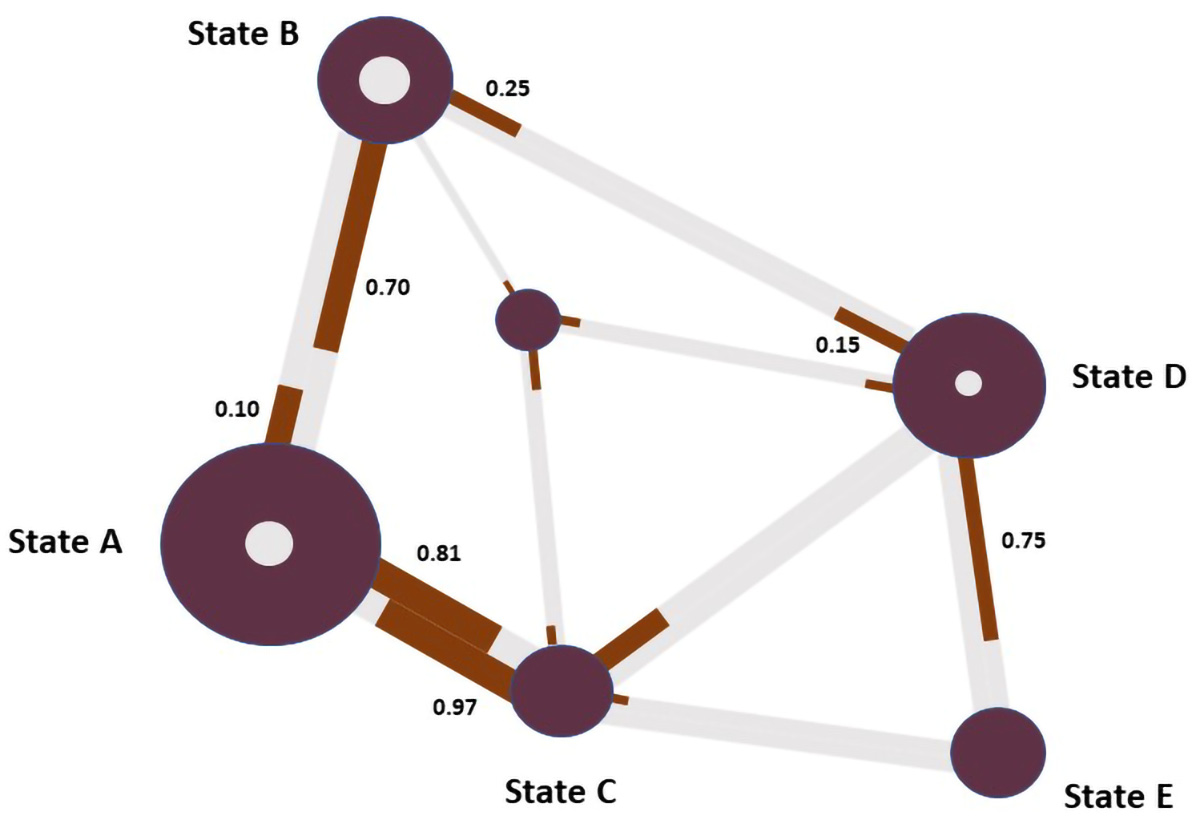
Mock state transitions among types of content in information patches.

Nodes of the network in Figure 6 represent types of patches (see: Comprehensive Codebook), and the dual edges between unique pairs of patch content types represent the transition probabilities between them. Each dual edge is made up of one edge representing the proportion of participants leaving a given patch (a transaction where the node/patch is called the ‘sending node’) and one edge representing the proportion of participants arriving at that patch (where the node/patch is called the ‘receiving node’). The proportion of participants transitioning to another patch is indicated by labeling each edge with the proportion and by treating each half of the dual edge as a relative frequency bar, coloring the proportion of the edge corresponding to the proportion of participants leaving the patch red instead of grey. Sometimes, all transitions between two states/nodes were unidirectional, in which case the corresponding half of the dual edge is completely colored grey. If there were no transitions between two given states/patches at all (i.e. bidirectionally), no edge is drawn at all. The size of each node represents the total number of participant-states spent at patches of the content type represented by the node. The size of the inner circle *within* each node is proportional to the number of times participants transition from one patch/state to another patch/state of the same content type, i.e. when a node is both the sender and the receiver in a given state transition. The larger the circle within the node, the higher the proportion of participant-state combinations that are self-references. Sometimes there are no self-references, in which case there is no circle within the node. The node size aesthetic can be mapped onto multiple data attributes (e.g., duration spent on a patch content type or the frequency of a content type within the sample / subsample). Figure 6 displays, for a given mock subsample for foraging, the transition probabilities among certain content types (dual edges), self-loops (circles within nodes), and in-patch foraging duration (node size).

**Figure 7.**
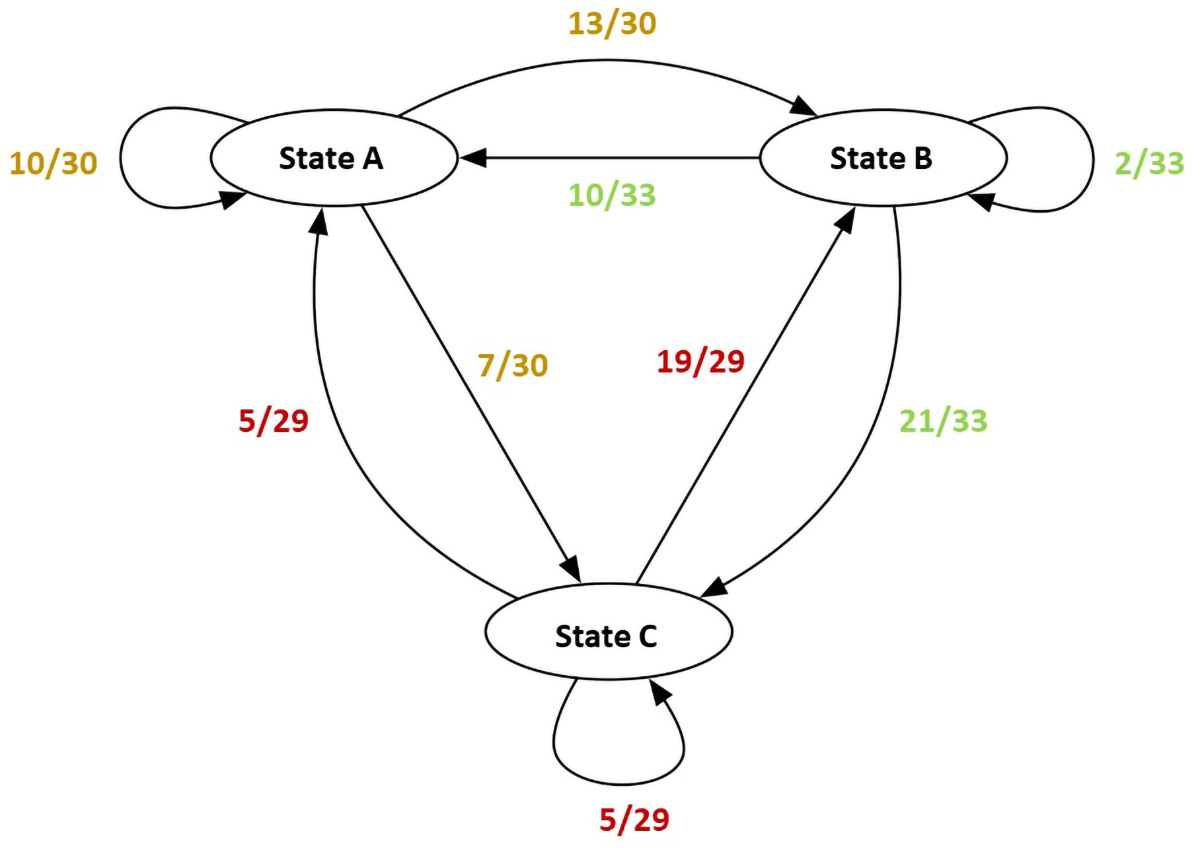
Mock state transition network and probabilities.

QUEST describes the transition states between steps (or states) in a process. The basic computations for QUEST are made with a state transition network where the frequencies of transitions from a particular state to itself and other states are added to compute the total transition counts for each state. Then, an adjacency matrix is created for each unit containing the transitions from each state to other states and to themselves. The matrix is asymmetric, that is, the transition probability from state A to state B may be different than the transition probability from B to A. The value is calculated by first summing all transition counts for a given state and then dividing the counts for a particular relationship (e.g., A to B) by the total count. This act generates the transition probability values, which fall between zero and one. Figure 7 shows a mock state transition network (left) with computed transition counts (right, top) and probabilities (right, bottom). Values in the state transition network are used to create the asymmetric adjacency matrix containing transition probabilities (gray cells represent self-loops). The contents of this matrix is then used as input for the network visualizer.

***RQ4: What patch features are utilized to assess the trustworthiness of (sources of) information?***

**Figure 8.**
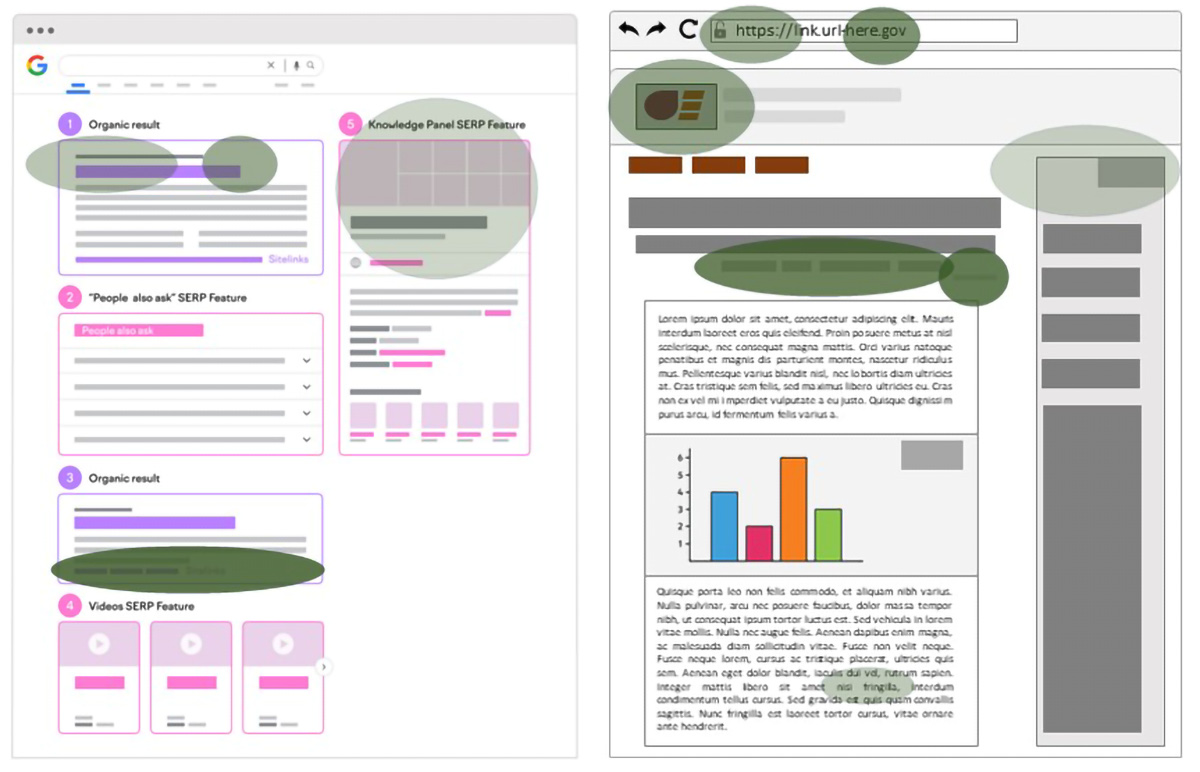
Mock patch features identified by participants during information appraisal.

Our fourth objective is to tally and visualize all structural elements of SERPs and websites that participants mention in their evaluation of information with which they are engaging ( *Appraisal* codes within the *Trustworthiness* code cluster). Figure 8 shows a mock-up of features a subsample identified ( *Appraisal* codes, green ovals); color saturation and alpha value represent code frequency. The more saturated and opaque the color, the higher the code frequency.

To obtain code frequencies, we will employ the rock R package. Frequencies will be computed for novices and experts separately. For each code, we will binarize occurrences per patch and sum those across patches in the 10-minute search task for every case. As participants foraged in a different number of patches, we will normalize code occurrences by dividing them by the number of patches visited. These normalized frequencies will be summed according to subsample to give us our final frequencies, which will then, in turn, be represented as colored ovals with various degrees of saturation juxtaposed onto schematic versions of both SERPs and websites.

***RQ5: How does information scent differ between novices and experts?***

To answer our last research question, we first develop a novel way of computing information scent, that is, participants’ assessment of a patch regarding its relevance to the task, its trustworthiness, and its ability to contribute to understanding the topic. We aim to compare novices’ and experts’ information scent throughout the duration of their search task.

As information scent is the participant’s representation of the extent to which a patch contributes to accomplishing their information goal, it is a function of participants’ processing and ultimate internal representation of the stimuli comprising each patch. However, we strive to distinguish stimulus properties from subsequent participant-specific attributes, such as perceptual and representational idiosyncrasies. Therefore, our analyses will involve both stimuli and participants’ representations of those. Thus, as determinants of information scent, we consider both the stimuli the participant encounter *and* the participants’ engagement with those stimuli in their think-aloud *or* their HCI. Stimuli contribute to higher information scent in the encountered patches if the participant engages with them, and to lower information scent if the participant fails to notice them. Conversely, if stimuli are absent, there is nothing to engage with (disregarding potential misperceptions for now) and thus do not contribute to information scent.

For this analysis, we are using all code clusters ( *Relevance*, *Trustworthiness*, and *Understanding*). All three data streams (stimuli, think-aloud, HCI) will be coded for both the presence and absence of constructs of interest. For example, a website feature (stimulus stream) may be “author affiliation” or “publication year”; participants may then interact with this part of the webpage (HCI stream) and/or mention it in the interview (think-aloud stream).

Codes are applied per data stream per patch. Then, code occurrences in the three code clusters are evaluated to create a new derived variable. The default value is set to 0. The variable is set to +1 for all patches where stimuli occurred (as represented by codes in the stimulus stream) and were engaged with (as represented by code presence in the narrative or HCI stream); it is set to -1 where stimuli occurred, but were not perceived by the participant (as represented by code absence in the narrative or HCI stream). Table 3 shows code co-occurrences per patch (rows). The columns in Table 3 are constituted by code designations in three data streams (1 for presence, 0 for absence of code), and the derived variable (possible values: -1, 0, +1), as well as a qualitative interpretation of the co-occurrence.

**Table 3.** Co-occurrences for code author affiliation (AuthAff) in stimulus data (STIM), think-aloud data (TA), and human-computer interaction data (HCI), as well as the derived variable from these co-occurrences.

Patch	STIM_ AuthAff_ present	STIM_ AuthAff_ absent	TA_ AuthAff_ present	TA_ AuthAff_ absent	HCI_ AuthAff_ present	HCI_ AuthAff_ absent	DERIVED- VARIABLE_ AuthAff	Qualitative explanation
1	1	0	0	0	1	0	+1	AuthAff present in stimulus and participant clicked on it
2	1	0	0	0	0	0	-1	AuthAff present in stimulus but participant did not engage with it in think-aloud or HCI
3	0	1	0	0	0	0	0	AuthAff absent in stimulus but participant did not mention this in think-aloud
4	0	1	0	1	0	0	0	AuthAff absent in stimulus and participant noted this in think-aloud

Information scent is the sum of values for each derived variable for all code clusters across patches. The values from derived variables are represented in columns of a matrix where rows are constituted by visited patches. Table 4 shows code co-occurrences as a derived variable for four codes (A-D) across five patches and the information scent computed for this fictional participant.

**Table 4.** Mock derived variables for patches 1-5 and information scent.

	Derived variable A	Derived variable B	Derived variable C	Derived variable D
Patch 1	0	0	0	-1
Patch 2	0	0	0	1
Patch 3	1	-1	1	1
Patch 4	0	1	0	1
Patch 5	1	1	-1	0
**Sum = .3**	**2**	**2**	**0**	**2**

In the example displayed in Table 4, out of a maximum information scent value of 20 for the visited patches, the participant received 6. To compute information scent, we divide the values accumulated across visited patches by the maximum information scent, which results in a value of .3 for this particular participant. Information scent can be computed for a participant at any point during a search or per visited patch. These can be represented in a line graph to show the evolution of information scent as the participant approaches the information goal. This mode of computation enables richer patches to have higher contributions to overall information scent, and also for overall informational scent in a task to be absolutely higher or lower (e.g., a PDF of an article can have a higher information scent than a flyer).

## Conclusions

Quantifying qualitative data, aligning data streams, and representing all information in a tabularized dataset allows us to group data according to various participant attributes and employ data visualization techniques to pinpoint patterns therein.

### Limitations

Although these quantitative models offer insights into patterns in large amounts of qualitative data, the techniques have limitations. ENA models display the relative frequency of co-occurrences between code pairs within designated segments of data; as of now it has no hypergraph capabilities (to e.g., display co-occurrence among triads). QUEST does not display transition probabilities between specific websites, as this would be unwieldly and uninformative on the subsample level. States, therefore, represent codes for content types, and transition probabilities can only be computed for two given states, not all states in a process (entire search task). This also implies that because content type is an inductively developed coding scheme, there are many feasible alternative schemes, which has an effect on results. To address this and test how strong these effects are, we intend to conduct sensitivity analyses for different potential coding schemes. Our operationalization of information patch is based on URL, which allows for dynamic content to differ across users, or even change within a 10-minute task. Also, often content may not be visibly accessed by participants, and thus not recorded as part of our data. This leads to only partial recordings of patch content and potentially allows the same URL to yield different content for users. This, in turn, means that the identifier given to patches based on URL may be misleading if the content does not match precisely, and may also influence coding in all three code clusters to some degree. In the future, this will be rectified by scraping patch content and running a comparison among patches with the same identifier to make sure their content matches.

### Intervention development

This feasibility study will enable us to better understand and map processes in data collection, curation, coding, segmentation, data stream alignment, and modelling. After modifying protocols according to these insights, we will be able to automate processes such as scraping screen content data, recording HCI, and some parts of coding and segmentation. This will allow us to conduct a larger-scale, multi-site study with a more clear and more automated workflow. Findings from this study and the subsequent one will be employed to create a digital intervention for vulnerable individuals, more specifically people who are active online but have low DHL. The intervention itself will be created in a way that will be able to accommodate novel results from future studies reproducing these methods.

## Data Availability

OSF. Smart Online Searching To Increase Patient Safety (SOS-TIPS) https://osf.io/ynt7a/. This project contains the following underlying data: Data_Attributes (Characteristics of data providers in YAML format) Data_HCL (Transcription of screen content according to our machine-readable template) Data_narratives (Transcription of retrospective think-alouds) Data are available under the terms of the Creative Commons Attribution 4.0 International license (CC-BY 4.0).

## Footnotes

^4^ LimeSurvey, for example, is a free, online alternative to Qualtrics.

^5^ Whisper by Openai, is a free alternative that can be utilized for the purpose of reproducibility.
