# Supplementary material for: A feasibility study for a unified, multimodal analysis of online information foraging in health-related topics
Source: Open Res Eur. 2024 Mar 6;3:98. Originally published 2023 Jun 20. [Version 2] doi: 10.12688/openreseurope.16119.2 (PMC11036032; doi:10.12688/openreseurope.16119.2)
Supplement: Peer review history

# Reviewer report: Reviewer response for version 2

**DOI:** 10.21956/openreseurope.18703.r38788

**Published:** 2024-04-22

**Authors:** Seung B. Lee

**Affiliations:** 1 Pepperdine University, Malibu, California, USA

Many thanks to the authors for their responses and the revisions to the manuscript. I have no further comments.

Are sufficient details provided to allow replication of the method development and its use by others?

Yes

Is the description of the method technically sound?

Yes

Are the conclusions about the method and its performance adequately supported by the findings presented in the article?

No

Is the rationale for developing the new method (or application) clearly explained

Yes

If any results are presented, are all the source data underlying the results available to ensure full reproducibility?

No source data required

Reviewer Expertise:

Collaborative learning, epistemic network analysis, STEM education

I confirm that I have read this submission and believe that I have an appropriate level of expertise to confirm that it is of an acceptable scientific standard.

# Reviewer report: Reviewer response for version 1

**DOI:** 10.21956/openreseurope.17402.r35571

**Published:** 2023-12-15

**Authors:** Ljerka Luić

**Affiliations:** 1 University North, Varaždin, Croatia

This article describes the design and planned analyses for a feasibility study on online information foraging of health-related content. In the introduction, the authors Zörgő at al. elaborated the semantics of digital health literacy, provide a thorough overview of the theoretical framework of the information scent and propose the following modifications of information seeking theory: (1) Code co-occurrences instead of semantic similarity, (2) Sampling from the target population, (3) Differences between individuals and (4) Differences over time. The article also provides detailed descriptions of the data collection and preparation processes, involving the observation, survey and interview. The article makes a strong case for the consideration of new methodological approaches to examine information foraging behavior in digital environments, particularly among individuals seeking health-related information. Furthermore, the article represents the contribution to the corpus of knowledge primarily in the field of information science, but it also has a significant value in an applicative sense in the context of application in the field of medicine.

 The article is well structured. The sessions follow the logic of presenting the themes described in the abstract well and their excerpts clearly describe the (subject) session. The research questions from the introduction summarized and very well elaborated. The idea is clearly described with defined methods, results and conclusions. Diagrams presented in the article are accompanied with matching well-structured textual description that enables their comprehension. Figures and tables are technically well executed and very well linked with the main text. The authors conducted a well-documented analysis of the data and included and conducted correct tests and methods as well as correct validation procedures. Suggestion, correct the amount of the sum in the column labelled “Derived variable B” in the Table 4. All references are adequate and recent.

 Concluding summary, the article contains innovative research methods and original approach to research. I do encourage the authors to carry on research work, expand their research questions and apply new innovative research methods.

Are sufficient details provided to allow replication of the method development and its use by others?

Yes

Is the description of the method technically sound?

Yes

Are the conclusions about the method and its performance adequately supported by the findings presented in the article?

Partly

Is the rationale for developing the new method (or application) clearly explained

Yes

If any results are presented, are all the source data underlying the results available to ensure full reproducibility?

No source data required

Reviewer Expertise:

Information and communication sciences: informatology, information systems, digital intelligence, digital literacy, digital communication

I confirm that I have read this submission and believe that I have an appropriate level of expertise to confirm that it is of an acceptable scientific standard.

# Response

**Published:** 2024-02-07

**Authors:** Szilvia Zörgő

**Affiliations:** Care and Public Health Research Institute, Maastricht University, Maastricht, Limburg, The Netherlands

Responses to Reviewer 2: Thank you for your feedback and for approving our first version. We have modified the value you indicated in the table, and appreciate the time and effort you sacrificed in reading and commenting on our work! It is very much appreciated!

# Reviewer report: Reviewer response for version 1

**DOI:** 10.21956/openreseurope.17402.r32976

**Published:** 2023-09-06

**Authors:** Seung B. Lee

**Affiliations:** 1 Pepperdine University, Malibu, California, USA

This article describes the design and planned analyses for a feasibility study on online information foraging of health-related content. It outlines novel methods for examining how individuals find, understand and evaluate health information in digital environments. Drawing upon information foraging theory, the authors propose several modifications to the current conceptualization, particularly in defining information scent as being comprised of both the stimuli and user attributes. The inclusion of user attributes—factors influencing a person’s ability to detect and process stimuli—into the definition makes it possible for information scent to vary across individuals and over time. In operationalizing information scent, the authors suggest the application of code co-occurrence over semantic similarity. The article also provides detailed descriptions of the data collection and preparation processes, involving the observation, survey and interview of 20 participants. Rather than providing the results from the analyses of collected data, only the analytical plans for each of the five research questions are presented along with visuals created from mock data. It concludes with a brief discussion on its limitations and implications for further study.

 Overall, the article makes a strong case for the consideration of new methodological approaches to examine information foraging behavior in digital environments, particularly among individuals seeking health-related information. The emphasis on the integration of user attributes into the operationalized definition of information scent brings it closer to its original conceptualization: “user behavior in the information environment is guided by information scent, which is determined from the perception of the value and cost of the information with respect to the goal of the user” (Chi et al., 2001, p. 490). From this perspective, the effort to more fully incorporate factors influencing the subjective perception of the user in the derivation of information scent can be seen as an enhancement to existing approaches focusing mainly on the stimuli.

 The data collection and preparation processes are meticulously and thoroughly presented. The results of the pre- and post-task surveys, including demographic information and task-related attitudes and opinions, are captured in the case attribute records. Recordings of observations and interviews are used to produce the three core streams of data: think-aloud, human computer interaction (HCI), and screen content. Given the multitude of steps involved in the data preparation process, it is helpful to see the transcribed and coded data for the three streams as displayed in Figure 3. Similarly, it would be useful to include a sample of the unified dataset as a separate figure to demonstrate how the three data streams as well as the attribute variables can all be aligned and placed within a single data table.

 With the overarching objective of the feasibility study to compare experts and novices to identify good practices and challenges in the seeking health-related information, the five research questions (RQs) are organized to gain insights on different aspects of the foraging process. The first four RQs each rely on a single stream of data. RQs 1, 2 and 4 analyze the think-aloud data, while RQ 3 uses patch type categorizations coded from the observation video data. RQs 1 and 2 use Epistemic Network Analysis (ENA) to model the co-occurrence of codes related to information scent. RQ 1 compares the ENA models of novices and experts and RQ 2 explores the changes in the ENA models (trajectories) over the duration of the search task. Sufficient descriptions and explanations of the ENA modeling process are provided in the manuscript to facilitate replication. While the stanza window type and size to be applied are not specified, the intention may have been to provide greater flexibility during the analysis for identifying the parameters most suitable for the collected data. In RQ 3, a new tool called Qualitative/Unified Exploration of State Transitions (QUEST) is proposed to explore the transition from one type of patch to the next. Two network diagrams (Figures 6 and 7) are used to display the transition probability between various types of patches/states. It could be useful to clarify how the two visualizations differ in their applications and when one should be used over the other. As for RQ 4, it is noted that the calculated final code frequencies will be “represented as colored ovals with various degrees of saturation juxtaposed onto schematic versions of both SERPs and websites.” Given that the code frequencies may be presented more precisely as numerical values, it would be helpful to articulate the rationale for adopting such a visualization.

 The multimodal nature of the data is fully utilized in the final research question (RQ 5), which asks how information scent differs between novices and expert information seekers. To address this question, the authors describe the process for computing a new composite score for information scent that brings together all three core data streams by synchronizing them through the use of patch as the unit of analysis. The resulting score is a value between 0 and 1 for each participant. It would be useful to articulate how the individual scores would be used to compare information scent between the groups of novices and experts. Furthermore, it appears that the computation of a composite score for information scent in this manner results in a severe flattening of the information that has been captured. For this reason, the analytical plan as currently described for RQ 5 may not enable researchers to thoroughly examine the question of “how” information scent is different between the two groups (beyond simply answering the question of whether they differ and by how much). While the possibility for conducting a more fine-grained analysis using this technique is mentioned (e.g. “information scent can be computed for a participant at any point during the search or per visited patch”), it would be helpful to elaborate how such types of analyses may be applied to take greater advantage of the meticulously coded/labeled data in addressing the research question in a more comprehensive manner. Lastly, a review of Table 4 is suggested. The values for the column labeled “Derived variable B” are 0, 0, -1, 1, and 1 for the five patches. It would seem that the sum of these values would be 1; however, the table currently indicates that it is 2.

**References**

 Chi, E. H., Pirolli, P., Chen, K., & Pitkow, J. (2001). Using information scent to model user information needs and actions and the Web. In *Proceedings of the SIGCHI Conference on Human Factors in Computing Systems (CHI '01) *(pp. 490-497). Association for Computing Machinery. https://doi.org/10.1145/365024.365325

Are sufficient details provided to allow replication of the method development and its use by others?

Yes

Is the description of the method technically sound?

Yes

Are the conclusions about the method and its performance adequately supported by the findings presented in the article?

No

Is the rationale for developing the new method (or application) clearly explained

Yes

If any results are presented, are all the source data underlying the results available to ensure full reproducibility?

No source data required

Reviewer Expertise:

Collaborative learning, epistemic network analysis, STEM education

I confirm that I have read this submission and believe that I have an appropriate level of expertise to confirm that it is of an acceptable scientific standard, however I have significant reservations, as outlined above.

## References

[1] : Using information scent to model user information needs and actions and the Web. In Proceedings of the SIGCHI Conference on Human Factors in Computing Systems (CHI '01). *Association for Computing Machinery* .2001; 10.1145/365024.365325 490-497 10.1145/365024.365325

# Response

**Published:** 2024-02-07

**Authors:** Szilvia Zörgő

**Affiliations:** Care and Public Health Research Institute, Maastricht University, Maastricht, Limburg, The Netherlands

Responses to Reviewer 1:

 Most importantly, thank you for the time and energy you have put into reviewing our article! Your comments were all very much appreciated, and our responses are listed below:

 Comment: "It could be useful to clarify how the two visualizations differ  in their applications and when one should be used over the other."

 Response: Thank you, this indeed was unclear. We have added: “Figures 6 and 7 are not coordinated, they merely serve as illustrations: Figure 7 is a visual representation of possible transition counts and probabilities, examples of those are provided in Figure 6.”

 Comment: "Given that the code frequencies may be presented more precisely as numerical values, it would be helpful  to articulate the rationale for adopting such a visualization." Response: Thanks for this comment! We have added: “Albeit information that QUEST visualizes could be presented as numerical values, we believe the network representation aids readability and conveyability of results and decreases the risk of bias due to the accuracy affordance carried by numerical representations.” as explanation.

 Comment: "It would be useful to articulate how the individual scores would be used to compare information scent between the groups of novices and experts. Furthermore, it appears that the computation of a composite score for information scent in this manner results in a severe flattening of the information that has been captured. For this reason, the analytical plan as currently described for RQ 5 may not enable researchers to thoroughly examine the question of “how” information scent is different between the two groups  (beyond simply answering the question of whether they differ and by how much)."

 Response: This is a very valuable comment, thank you! We neglected to state the following, which has now been added: “We will also generate heat maps so individual and group differences can be inspected for every unique code alignment (see: Table 4).”

 Comment: "it would be helpful  to elaborate how such types of analyses may be applied to take greater advantage of the meticulously coded/labeled data in addressing the research question in a more comprehensive manner. Lastly, a review of Table 4 is suggested. The values for the column labeled “Derived variable B” are 0, 0, -1, 1, and 1 for the five patches. It would seem that the sum of these values would be 1; however, the table currently indicates that it is 2."

 Response: Thank you so much for catching that, you are completely correct; we have modified the table.
